# The Effectiveness of Wearable Devices Using Artificial Intelligence for Blood Glucose Level Forecasting or Prediction: Systematic Review

**DOI:** 10.2196/40259

**Published:** 2023-03-14

**Authors:** Arfan Ahmed, Sarah Aziz, Alaa Abd-alrazaq, Faisal Farooq, Mowafa Househ, Javaid Sheikh

**Affiliations:** 1 AI Center for Precision Health Weill Cornell Medicine-Qatar Doha Qatar; 2 Qatar Computing Research Institute Hamad Bin Khalifa University Doha Qatar; 3 College of Science and Engineering Hamad Bin Khalifa University Doha Qatar

**Keywords:** diabetes, artificial intelligence, wearable devices, machine learning, blood glucose, forecasting, prediction

## Abstract

**Background:**

In 2021 alone, diabetes mellitus, a metabolic disorder primarily characterized by abnormally high blood glucose (BG) levels, affected 537 million people globally, and over 6 million deaths were reported. The use of noninvasive technologies, such as wearable devices (WDs), to regulate and monitor BG in people with diabetes is a relatively new concept and yet in its infancy. Noninvasive WDs coupled with machine learning (ML) techniques have the potential to understand and conclude meaningful information from the gathered data and provide clinically meaningful advanced analytics for the purpose of forecasting or prediction.

**Objective:**

The purpose of this study is to provide a systematic review complete with a quality assessment looking at diabetes effectiveness of using artificial intelligence (AI) in WDs for forecasting or predicting BG levels.

**Methods:**

We searched 7 of the most popular bibliographic databases. Two reviewers performed study selection and data extraction independently before cross-checking the extracted data. A narrative approach was used to synthesize the data. Quality assessment was performed using an adapted version of the Quality Assessment of Diagnostic Accuracy Studies-2 (QUADAS-2) tool.

**Results:**

From the initial 3872 studies, the features from 12 studies were reported after filtering according to our predefined inclusion criteria. The reference standard in all studies overall (n=11, 92%) was classified as low, as all ground truths were easily replicable. Since the data input to AI technology was highly standardized and there was no effect of flow or time frame on the final output, both factors were categorized in a low-risk group (n=11, 92%). It was observed that classical ML approaches were deployed by half of the studies, the most popular being ensemble-boosted trees (random forest). The most common evaluation metric used was Clarke grid error (n=7, 58%), followed by root mean square error (n=5, 42%). The wide usage of photoplethysmogram and near-infrared sensors was observed on wrist-worn devices.

**Conclusions:**

This review has provided the most extensive work to date summarizing WDs that use ML for diabetic-related BG level forecasting or prediction. Although current studies are few, this study suggests that the general quality of the studies was considered high, as revealed by the QUADAS-2 assessment tool. Further validation is needed for commercially available devices, but we envisage that WDs in general have the potential to remove the need for invasive devices completely for glucose monitoring in the not-too-distant future.

**Trial Registration:**

PROSPERO CRD42022303175; https://tinyurl.com/3n9jaayc

## Introduction

### Background 

Despite advances over the past decades, including improved life expectancy and quality of life [1], in 2021 alone, diabetes mellitus, a metabolic disorder primarily characterized by high blood glucose (BG) levels, affected 537 million people globally. According to the International Diabetes Federation, over 6 million deaths were reported. These staggering figures are projected to increase in the coming years, with forecasts predicting that 643 million people (1 in 9 adults) will have diabetes by 2030. Furthermore, it is estimated that 784 million people will have diabetes mellitus by 2045 [2]. For people living with diabetes, maintaining a normal range of BG levels is vital; otherwise, short- or long-term complications can occur due to hyperglycemia or hypoglycemia. The risk of cardiovascular-related disease is dramatically increased if a higher than optimal BG level is observed, which could ultimately lead to death. Complications can also lead to heart attacks, strokes, loss of vision, kidney failures, and nerve damage, as well as complications during pregnancy. The World Health Organization outlines the need for collaborative intervention from various stakeholders, such as health care providers, governments, medicine suppliers, and food suppliers, along with the technology industry, which is seen as a key component, for there to be a significant impact in the reduction of diabetes [3].

Despite breakthroughs in BG monitoring techniques, most detection technologies remain invasive. The commonly used home electronic glucose meters need the person with diabetes to invasively self-prick to extract blood from the fingertips of the person with diabetes, exposing the person with diabetes to infections as well as stress and suffering caused by the recurrent operation, which is often expected numerous times per day. The availability and improvements of smart gadgets such as smartphones have made diabetes-related functions more accessible. Many studies have been conducted to investigate this much-appreciated technology [4,5]. These often still necessitate the use of an externally attachable sensor, and monitoring is then given via an app or a separate continuous glucose monitoring device, which is often semi-invasive and requires connectivity range via Bluetooth or Wi-Fi signal. The use of completely noninvasive technologies, such as wearable devices (WDs), to regulate and monitor BG levels in people with diabetes is a relatively new concept and still in its infancy. Researchers have reported on the efficacy of sensors in commercially accessible products such as smart watches and smart bands in diabetes monitoring [6,7].

When used properly, these technologies are affordable and easily accessible, and they can improve the quality of life of patients noninvasively. Due to their wide adoption and acceptance globally, researchers and patients have a unique opportunity to leverage WDs for the purpose of providing noninvasive medical care away from hospital settings in a portable yet affordable manner. Even though WDs do not possess the capabilities of smartphones, they are increasingly able to gather, store, transmit, and process data, the features of which can be applied for management, treatment, assessment, forecasting (based on past observations), and even prediction (taking associated data into consideration, eg, diet, activity, and medications along with previous BG values), the latter 2 terms often used interchangeably. For the purpose of this study, neither did we distinguish these terms in our search and filter processes as this is how we found their usage in the reviewed studies, nor did we attempt to classify them according to these definitions. Many WDs are often linked through Wi-Fi or Bluetooth to external devices such as smartphones, where computationally intensive processing is conducted for the simple purpose of storage or as a gateway to cloud spaces. Cloud storage allows physicians to monitor patients without requiring hospitalization. Several valuable sensors are already integrated into WDs like smartphones, including near-infrared (NIR) accelerometer, galvanic skin response, electrocardiogram, and photoplethysmography (PPG) sensors. Due to WDs being in close contact with the user, they provide further advantages over external sensor-driven devices when it comes to sensing physiological signs such as skin temperature and heart rate. This is particularly interesting for forecasting and monitoring diabetes-related metrics.

To digest meaningful knowledge from the large amounts of continuous data generated by WDs, artificial intelligence (AI) algorithms are used to provide advanced and clinically meaningful analytics. Machine learning (ML) as a terminology is often used interchangeably with AI, although technically it is a subset of AI. As a broad definition, AI is when machines are made smarter, and ML is a set of AI algorithms that learn patterns from data while having the ability to self-learn; over time, they get ever smarter without human intervention. Deep learning is a further branch of AI, which processes large amounts of data using neural networks that are computational models mimicking the human brain. There are 2 types of ML algorithms classifications: classical and modern. In comparison to modern approaches, classical methods require less training data and computer resources for pattern recognition. Modern approaches, on the other hand, frequently outperform traditional ones. Deep learning is a modern ML methodology in which algorithms replicate the brain’s neural networks to train with or without supervision; yet, unlike classical ML approaches, which are easy to explain, it might suffer from the “black box” problem. While some researchers have produced prototypes specifically designed with diabetes in mind, many have taken existing WDs not originally designed for diabetes management and adapted them by changing the sensory data in order to use them for diabetes-related metrics [8,9]. WDs have a wide range of uses, including forecasting, diagnostics, glucose estimation, monitoring, prevention, and classification. Unfortunately, the reported studies are still low compared to that of smartphones. By using ML algorithms from the expanding field of AI and correctly managing and processing enormous amounts of data, there is untapped potential to improve the quality of life for those with diabetes.

### Research Gap and Aim

Several studies have explored the use of WDs that use ML models for forecasting BG levels, but the evidence from these studies is scattered. Existing studies may also have different scopes and various aims, and therefore, systematic reviews are needed to aggregate the available evidence and draw conclusions about their effectiveness. A recent systematic review looked at mobile and wearable technology for monitoring parameters related to diabetes mellitus; although the authors report some ML features of each review, it does not include in-depth extraction of features (ie, the focus is not on AI) [10]. Furthermore, the same study does not report metrics related to the performance of ML algorithms used within each reviewed study, such as accuracy, Clarke grid error (CGE), and root mean square error (RMSE). Another recent systematic review does report outcome metrics such as sensitivity and accuracy. Although their review contains WDs within, the focus was on any technologies using deep learning for diabetes care, therefore no in-depth ML-related features were extracted [11]. Other recent reviews in this field are not detailed systematic reviews that focus on using ML; rather, they discuss the development of WDs for glucose biosensing [12] or the current status and challenges with available devices [13]. To address these limitations, this review aims to examine the effectiveness of WDs that use ML models for the purpose of BG-level forecasting. To the best of our knowledge, this is the first systematic review covering this topic.

## Methods

### Study Registration

This systematic review was conducted following the Preferred Reporting Items for Systematic Reviews and Meta-Analyses (PRISMA) guidelines [14]. The protocol has been registered with the International Prospective Register of Systematic Reviews (PROSPERO; ID: CRD42022303175).

### Search Strategy

#### Search Sources

To identify relevant studies, 6 electronic databases were searched: MEDLINE, PsycInfo, Embase, IEEE Xplore, ACM Digital Library, and Web of Science. Google Scholar was used to identify further grey literature. We inspected the first 100 hits retrieved by searching Google Scholar, as it sorts by relevance according to the search topic, typically returning several hundred items. The bibliographic collection took place from October 25, 2021, to October 30, 2021. The reference lists of the included articles were then searched for additional sources. The relevant papers that cited the included studies using Google Scholar’s “cited by” tool (forward reference list checking) were also checked.

#### Search Terms

Keywords were compiled by authors according to each database term; for example, IEEE and Google Scholar limit search queries were truncated based on their allowed limits. We applied a combination of keywords, “Diabetic” OR “Diabetes” describing the relevant population (diabetes), with each kind of relevant intervention to wearables (“wearable*” OR “smart watch*” OR smartwatch* OR “fitness band*” OR “flexible band*” OR “wristband*” OR “smart insole*” OR “bracelet*”) and AI (“Artificial Intelligence” OR “Machine Learning” OR “Deep Learning” OR “Decision tree” OR “K-Nearest Neighbor*” OR “Support vector machine*” OR “Recurrent neural network*” OR “convolutional neural network*” OR “Artificial neural network*” OR “Naïve Bayes” OR “Naive Bayes” OR “Fuzzy Logic” OR “K-Means” OR “Random Forest” OR “LSTM” OR “autoencoder” OR “boltzmann machine” OR “deep belief network”). For example, the following search term was applied in Google Scholar: (“Artificial Intelligence” OR “Machine Learning” OR “Deep Learning” OR “convolutional neural network*” OR “Artificial neural network*”) AND (wearable*” OR “smart watch*” OR “smart*”) AND (“Diabetic” OR “Diabetes”). A search time limit was set within the query from 2015 to the present, and the language in each database was set to English only.

#### Study Eligibility Criteria

The eligibility of the retrieved studies was checked against the criteria shown in Textbox 1. We included peer-reviewed articles and proposals that were about AI-driven WDs used by individuals for forecasting BG outside of a clinical setting. AI for the purpose of diabetes was a key criterion for inclusion, and the process had to be noninvasive. Refer to Textbox 1 for inclusion and exclusion criteria.

**Inclusion and exclusion criteria.**

**Inclusion criteria**

- Publications that are in English and published in 2015 and onwards
- Peer-reviewed articles and proposals
- Population with, or suspected to have, diabetes. No restrictions regarding age, gender, and ethnicity
- Commercial, medical, or prototypes but with condition wearable device and uses artificial intelligence (AI)
- Wearable useable by individual person not with the help of clinical staff or plugged in to hospital setting
- Wearables using methods for diabetes analysis are to be noninvasive
- Empirical studies looking at blood glucose levels in diabetes

**Exclusion criteria**

- Publications that are not in English, published before 2015, and not peer-reviewed
- Nondiabetic-related population
- Any study that does not contain AI as an intervention
- Not a wearable device (eg, artificial implant or body-infused device)
- Studies that include only statistical measures for the analysis of collected data
- Sensors or tracking devices infused inside a person’s body
- Wearable devices that need professional or hospital sittings

### Selection Process

For study selection, 2 reviewers (first and second author) having identified and removed duplicates independently reviewed the titles and abstracts of all retrieved papers. In the second phase, the reviewers read the full texts of the papers included in the first step. All the articles acquired from databases in a Research Information Systems format were uploaded to Rayyan software (Qatar Computing Research Institute, Hamad Bin Khalifa University) [15], a web-based tool for data management of systematic reviews. Rayyan was used to filter citation management. Throughout the process, any disagreements between the 2 reviewers were resolved through consensus via discussion and a third reviewer (third coauthor). To examine interrater agreement among reviewers, Cohen κ [16] was computed, and it was 0.88 and 0.91 in the first and second steps of the selection procedure, respectively, suggesting a very excellent degree of agreement.

### Data Collection Process

A data extraction form was designed by the first and second authors, as shown in Multimedia Appendix 1. The same 2 authors extracted the data using MS Excel, and any discrepancies were resolved by discussion and agreement.

### Study Risk of Bias (Quality) Assessment and Concerns of Applicability

Two reviewers independently assessed the risk of bias of the included using an adapted version of the Quality Assessment of Diagnostic Accuracy Studies—Revised (QUADAS-2) tool [17]. A checklist was compiled after consulting other similar study approaches [18-20]. A third reviewer resolved disagreements between both reviewers. This tool is intended for use in systematic reviews to assess the risk of bias and applicability of primary diagnostic accuracy studies. The quality of chosen publications was assessed using the QUADAS-2 criteria, which evaluated four domains: (1) patient selection, (2) index test, (3) reference standard, and (4) flow and timing. As shown in Table 1, the signaling questions for each QUADAS-2 domain were adapted to the purpose of this evaluation. For each domain, this evaluation gave a risk of bias to research and ranked it as low (score=2), high (score=1), or unclear (score=0). Each study’s total score was computed by summing the number of satisfied criteria for each signaling question following under respective domains, with a higher score reflecting greater methodological quality. The 2 independent reviewers (authors 1 and 2) piloted the adapted QUADAS-2 tool (a checklist was formed after consultation with similar papers) before applying it to the selected studies (12 articles), and disagreements were addressed by consensus.

**Table 1:** Quality Assessment of Diagnostic Accuracy Studies-2 criteria used for qualitative assessment description.

Patient selection	Index test	Reference standard	Flow and timing
Signaling question 1: Data preprocessing specified?	Signaling question 1: Data acquisition methods detailed?	Signaling question 1: Is the reference standard likely to correctly classify the target condition?	Signaling question 1: Was there an appropriate interval between index tests and reference standard?
Signaling question 2: At least 50 participants were selected for analysis	Signaling question 2: BG^a^-level forecasting or prediction approach detailed? That is, network architecture provided or ML^b^ models parameters?	Signaling question 2: Were the reference standard results interpreted without knowledge of the results of the index test?	Signaling question 2: Did all patients receive a reference standard?
Signaling question 3: Balance of number of participants within the subgroups (ie, diabetic or nondiabetic, male or female, healthy or some disease)	Signaling question 3: More than one performance metrics used	Signaling question 3: Sufficient detail to allow replication about definition of ground truth (Was the method described in sufficient detail to reproduce the presented results?)	

^a^BG: blood glucose.

^b^ML: machine learning.

### Data Synthesis Methods

The narrative technique was used to synthesize the data from the included research. Narrative research is a broad term that encompasses a wide range of methods that rely on people’s written or spoken words, as well as visual representations [14]. Study information and data were synthesized by the second author from an Excel data extraction sheet related to the characteristics of recognized studies meeting the inclusion or exclusion criteria. The focus of the analysis was on studies that make use of AI and ML technologies for diabetes patients’ management and handling via WDs for BG level forecasting or prediction.

A traditional meta-analysis was not possible due to (1) the paucity and lack of raw data required to meta-analyze accuracy measures and (2) the considerable clinical and methodological heterogeneity in the included studies in terms of characteristics of WDs (eg, WD type, placement of the WD, device brand, and sensing approach), AI algorithms (eg, ML category, data size, data type, and type of validation), and performance metrics (eg, accuracy, mean absolute deviation, RMSE, and Clarke grid error). Due to this, we were unable to comment on pooled metrics. Previous systematic reviews looking at the application of AI also reported similar reasoning [19,21].

## Results

### Study Selection

**Figure 1:**
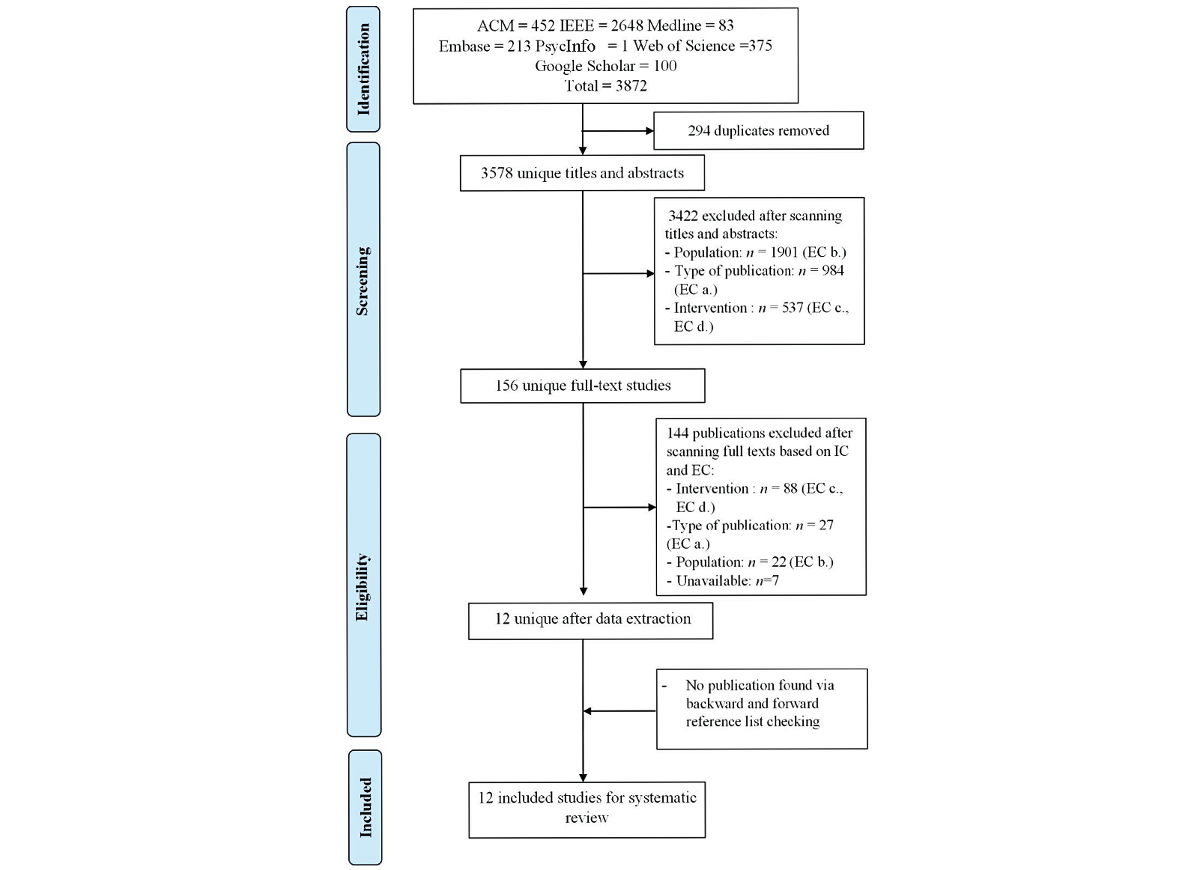
Preferred Reporting Items for Systematic Reviews and Meta-Analyses chart. IC: inclusion criteria; EC: exclusion criteria.

Having searched 7 bibliographic databases, this study returned 3872 citations. As shown in Figure 1, we subsequently removed 294 duplicates. Further, 3422 records were excluded after checking their titles and abstracts for the reasons reported in Figure 1. Of the remaining 156 references, 144 publications were excluded during the full-text screening. With 12 studies remaining, this number remained unchanged even after performing backward and forward reference list checking. The final synthesis includes 12 studies that met our inclusion criteria. Figure 1 illustrates the PRISMA process that was followed.

### Study Characteristics

Table 2 shows that 6 of the included articles were published in 2019, whereas 2018, 2020, and 2021 each have 2 publications. The countries of Asia mainly Bangladesh, China, Pakistan, India, Sri Lanka, and Taiwan had the most publications (n=8, 66%), followed by North America, which included the United States and Mexico (n=3, 25%), and Europe, comprising Switzerland, had single study. The number of participants was reported in 10 studies and ranged from 2 to 514 subjects, with an average of 77 subjects. The diversity in participants was observed in a number of publications, such as the selection of people with diabetes in 42% (n=5) or gender differences in 50% (n=6) of studies. Half of the studies (50%) specified the age range of included participants, and all of them contained participants in the age range of 18-50 years. The duration of data collection from participants varies from study to study, with a minimum duration of 8 seconds and a maximum duration of 7 months.

**Table 2:** Study metadata and population characteristics.

Study	Year	Country	Participants, n	Male, n	Medical conditions	Age (years)	Duration of data collection
Hina et al [22]	2020	Pakistan	200	112	Diabetics	18-71	N/A^a^
Alfian et al [23]	2018	Switzerland	71	N/A	Diabetics	NR^b^	Months
Alarcon-Paredes et al [24]	2019	Mexico	514	N/A	N/A	18-44	Minutes
Islam et al [8]	2019	Bangladesh	25	N/A	Diabetics	22-25	Days
Kularathne et al [25]	2019	Sri Lanka	50	NR	N/A	40-50	N/A
Joshi et al [26]	2020	India	46	26	N/A	24-50	Seconds
Zhou et al [27]	2019	China	8	4	Diabetics	NR	Weeks
Mahmud et al [28]	2019	Bangladesh	15	12	N/A	NR	Minutes
Bent et al [29]	2021	United States	16	7	Prediabetics	35-65	Days
Lee et al [9]	2021	Taiwan	N/A	N/A	N/A	NR	N/A
Shrestha et al [30]	2019	United States	N/A	N/A	N/A	NR	Seconds
Li et al [31]	2018	China	2	N/A	N/A	NR	N/A

^a^NA: not applicable.

^b^NR: not reported.

### Quality Assessment Results

#### Risk of Bias in Studies

**Figure 2:**
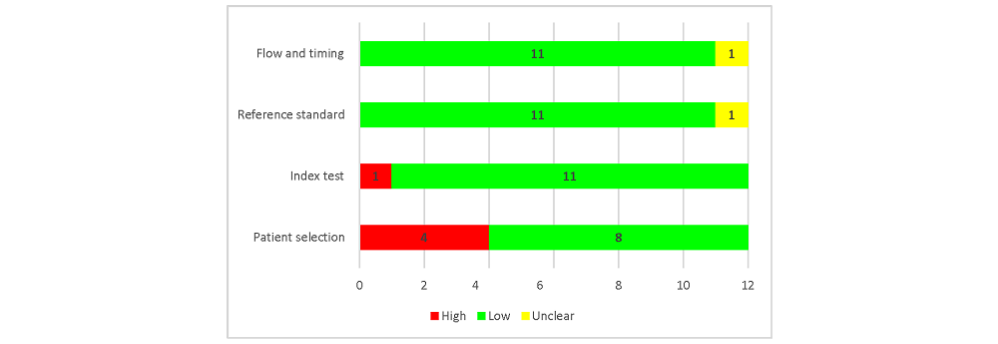
Risk of bias assessment.

In the patient selection domain, over one-third of the studies (n=6, 38.5%) reported a high risk of bias in patient sampling as they did not use an appropriate sampling process to select diverse participants among different subgroups. Incomplete coverage of data processing measures were taken for the conversion of WD-attained data as input to AI models (Figure 2). In most studies, the sample size was less than adequate for training and testing algorithms, which minimizes the impact of overfitting and enhances the quality performance metrics [32]. The risk of bias in index tests was rated as low in most studies (n=11, 92%), due to proper documentation of the nature of the tests, where the models’ data acquisition process, network architecture details, forecasting methodologies, and reasons were specified. The index test was evaluated on multiple performance metrics, thus better signifying the model prediction accuracy. Most of the studies used medically approved invasive methods for diabetes measurements as a reference standard, but one did not specify the details, which led to an unclear risk of bias. The reference standard in all studies overall (n=11, 92%) was classified as low as all ground truths were easily replicable. Since the data input to AI technology was highly standardized and there was no effect of flow or time frame on the final output, both factors were categorized in a low-risk group (n=11, 92%) except for one study that did not specify details and another that used a subset of participants and only considered the availability of better results if a specific medical condition (low BG level) was targeted. Multimedia Appendix 2 shows the QUADAS-2 tool risk of bias judgment in each included study across all 3 domains as well as applicability concerns for each study.

#### Concerns of Applicability

**Figure 3:**
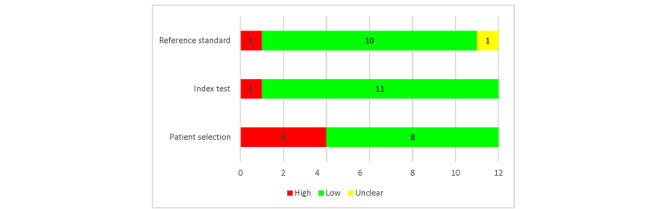
Concerns of applicability.

Figure 3 demonstrates the applicability concern in the patients’ selection domain. It was considered high in the majority of included studies (n=4, 33%) as the patient’s characteristics and the condition and setting of each test do not match the review question and criteria. Some studies selected participants randomly without checking their diabetic conditions, while some selected patients from other medical illnesses without considering the target audience. Similarly, in the index test, the included studies were deemed to have a low applicability issue. This is because the AI algorithm approach used in the included research corresponds to the review definition of AI. However, the reference standard’s applicability was assessed as unclear and high in single studies because the data samples in these studies were acquired from various databases without detailing selected conditions. Multimedia Appendix 3 demonstrates the details regarding each domain.

### Features of WD

**Figure 4:**
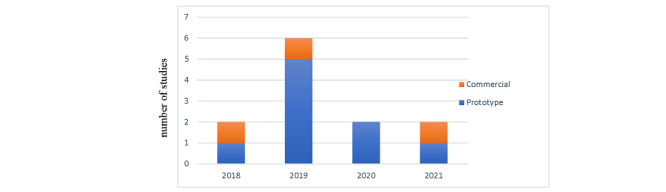
Evolution of wearable technology type.

Most studies developed their prototype (n=9, 75%), whereas only 25% (n=3) made use of commercially available WDs (Figure 4). As shown in Table 3, the most common type of WD used in the included studies was wearable sensors at 58% (n=7). More than half of the WDs were wrist-worn (n=7, 58%), followed by finger-worn (n=3, 25%). The Raspberry Pi Zero device brand was the most popular among studies (n=3, 25%). The sensing approach opted by most of the devices was opportunistic (n=7, 58%), where minimal to no input data were required from the participant’s side and sensors automatically collected data, while others (42%, n=5) used a participatory approach, where users’ input was exclusively required. Most of the studies used a single WD (n=10, 83%). The sensing technology type used in WDs was either built-in devices or comprised of multiple sensors incorporated in more than 1 device. Among the sensors used, NIR sensing was the most used (n=5, 42%) in combination with other sensors, followed by PPG sensor usage (n=5, 42%). The smartphone was the most common (n=6, 50%) gateway device used in studies for transferring data from WDs to end-host devices. The mode of data transfer between end points was mostly Bluetooth (n=5, 42%), followed by internet technology (n=3, 25%) consisting of either Wi-Fi signals or cellular networks; 5G technology was also observed. The end data host device (ie, where data were processed or stored) was cloud services, in half of the studies (n=6, 50%).

**Table 3:** Features of wearable devices used for blood glucose forecasting or prediction.

Authors	Wearable technology type	Wearable device type	Placement of wearable device	Device technology or brand	Sensing approach		Devices, n		Sensing type			Gateway		Host device	Mode of data transfer	
Hina et al [22]	Prototype	Wearable sensor	Finger	N/A^a^	Participatory		1		NIR^b^, PPG^c^		N/A			N/A	N/A	
Alfian et al [23]	Commercial	Smart wristband	Wrist	Mi Band 2: Mi Band 2, Mi Smart Scale, Caresens, and Omro	Participatory		4		BLE^d^ sensors		Smartphone			Cloud	Bluetooth	
Alarcon-Paredes et al [24]	Prototype	Wearable sensor	Hand	N/A	Participatory		1		PPG sensor and GSR^e^ sensor		N/A			N/A	N/A	
Islam et al [8]	Prototype	Wearable sensor	Finger	Raspberry Pi Zero	Participatory		1		Raspberry Pi camera		N/A			Smart devices	Internet	
Kularathne et al [25]	Prototype	Wearable sensor	Foot	Raspberry Pi Zero	Opportunistic		1		Tilt switch sensor, calibrated load cell sensor, DHT22^f^		N/A			Smart devices	Internet	
Joshi et al [26]	Prototype	Wearable sensor	Finger	N/A	Opportunistic		1		NIR		Smartphone			Cloud	Internet	
Zhou et al [27]	Commercial	Smart watch	Wrist	Glutrac	Opportunistic		1		NIR and ECG^g^		Smartphone			Cloud	Bluetooth	
Mahmud et al [28]	Prototype	Smart watch and wearable sensor	Wrist	Arduino Nano, Raspberry Pi	Opportunistic		2		PPG, temperature, GSR sensors		N/A			Raspberry Pi	Wired	
Bent et al [29]	Commercial	Smart wristband	Wrist	Empatica E4	Opportunistic		1		PPG, ACC^h^, EDA^i^, or GSR sensor, infrared thermopile		Smartphone or PC			Cloud	Bluetooth	
Lee et al [9]	Prototype	Smart watch	Wrist	Custom	Opportunistic	1		PPG and NIR		Smartphone			Cloud			Bluetooth
Shrestha et al [30]	Prototype	Smart wristband	Wrist	N/A	Participatory	1		Metal oxide semiconductor–based chemical sensors		Smartphone			Cloud			Bluetooth
Li et al [31]	Prototype	Wearable sensor	Wrist	Custom	Opportunistic	1		NIR		N/A			None			N/A

^a^N/A: not applicable.

^b^NIR: near-infrared.

^c^PPG: photoplethysmography.

^d^BLE: Bluetooth low energy.

^e^GSR: galvanic skin response.

^f^DHT22: digital-output relative humidity and temperature sensor.

^g^ECG: electrocardiogram.

^h^ACC: accelerometer.

^i^EDA: electrodermal activity.

### AI-Related Features of WDs

**Figure 5:**
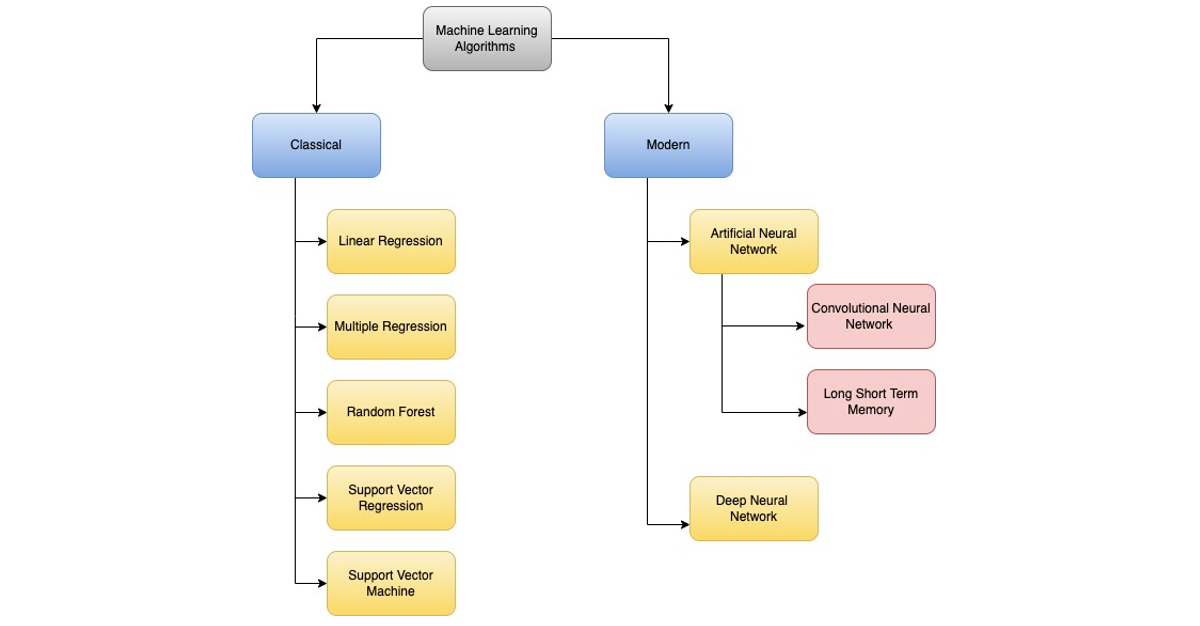
Hierarchical categorization of machine learning approaches.

A hierarchical categorization of the ML approaches was used in the chosen research; Figure 5 illustrates this. We observed that classical ML approaches were deployed by half of the studies (n=6, 50%) of those who mostly opted for ensemble-boosted trees mainly comprising random forest (n=5, 42%). In the majority of modern approaches, artificial neural networks–type convolutional neural networks (n=3, 17%) were used.

The input data used for ML models by a quarter (n=3, 25%) of the publications were BG levels, PPG signals, or NIR. Among all the other included articles, Shrestha et al [30] did not disclose the input details of the model. The validation method used by most of the studies was train or test split (n=8, 67%). The data decomposition of data sets used in studies was done based on the number of samples selected; the majority of the studies have separate data samples collected for testing purposes (n=5, 42%). The best models identified among studies from classical models were random forest (n=3, 25%) and convolutional neural network (n=3, 25%) in modern. Multiple and varied evaluation metrics were reported by studies, and the evaluation outcomes of the corresponding best models in each study are reported in Table 4. The most common evaluation metric used was CGE (n=7, 58%), followed by RMSE (n=5, 42%). However, owing to the lack of a uniform assessment metric across research, we do not summarize the reported metrics (calculation of mean, SD, etc). To validate the performance of ML models for forecasting or predicting BG levels from wearable data collected, all the studies made use of at least 1 ground truth method for reference glucose measurement. More than half of the studies made use of medical devices (med-devices; n=10, 83%) such as, glucometer or any portable device method used in daily routine. Other options used for ground truth collection were medical tests that may comprise laboratory blood tests or medical examinations, which were used by 25% (n=3), and an expert opinion was opted for in one of the studies [23].

**Table 4:** Artificial intelligence–related features of wearable devices for forecasting.

Author	Machine learning category	Algorithm used for forecasting or predictions	Input	Data validation method	Data set decomposition	Algorithm best performance	Reported best diagnostic performance of model	Ground truth
Hina et al [22]	Classical	Linear regression, FGSVR^a^, SVR^b^, and ensemble-boosted trees	PPG^c^ signal	Train or test split	Training: 60% of 200 subjects Validation: 40%	FGSVR	Coefficient of determination (R^2^): 0.937 mARD^d^: 7.62% RMSE^e^: 11.20 mg/dL Clarke error grid: 95%	Med-device
Alfian et al [23]	Modern	LSTM^f^	Insulin dose, BG^g^ level, meal ingestion, and exercise activity	Train or test split	Two BG data sets: Data set 1: 148 records CGM^h^ data set: single diabetes patient taken: 26,167 records Both data sets used 80% for training and rest for testing	LSTM	Correlation coefficient (r) and RMSE (Data set 1) RMSE: 25.621 mg/dL, r:0.647 (Data set 2) RMSE: 2.285 mg/dL, r: 0.999	Expert and med-device
Alarcon-Paredes et al [24]	Modern	ND^i^—ANN^j^	Fingertip images	K-fold cross validation, train or test split	Training: 514 hostograms Train or test (for model selection): 70% of whole data set Validation subset (model validation): 30%	ANN	MAEM^k^: 10.37 Clarke grid error: 90.32%	Medical
Islam et al [10]	Modern	CNN^l^	PPG signal, GSR^m^, and BG	Train or test split	210 data points Training: 204 data points Testing: 6 data points (4 nondiabetic and 2 diabetic)	CNN	Clarke grid error: 80%	Med-device
Kularathne et al [25]	Classical	Linear regression	Age, BMI, current blood glucose level, genetic factors, smoking, HbA1c,Carbs	NA^n^	NA	Linear regression	MSE^o^: 0.0150 R^2^ score: 0.7834 Variance score: 0.7346	Medical and med-device
Joshi et al [26]	Classical and modern	Deep neural network, MPR^p^	NIR^q^ signals	Train or test split	Training samples: 187 Testing samples: 46	MPR3	mARD: 4.86% AvgE: 4.88% Mean absolute deviation: 9.42% RMSE: 13.57 mg/dL	Medical and med-device
Zhou et al [27]	Classical	RF^r^	NIR signals, heart rate variability, pulse transfer time, BG level	Train or test split	Training samples: ND Test samples: 168	RF	Clarke grid error: 80.35% Avg RMSE: 1.44 mg/dL	Med-device
Mahmud et al [28]	Modern	CNN	Infrared channels, GSR and temperature signal	Train or test split	Training samples: 15 instance data of 15 subjects Testing: another 25 data Chosen data length: 1024	CNN	Clarke grid error (values not mentioned)	Med-device
Bent et al [29]	Classical	Multiple regression model, RF	Interstitial glucose summary and glucose variability metrics	Train or test split, leave on out	Training: 16 participants Testing: 10 participants	RF	RMSE: 35.7 mg/dL Mean absolute percentage error: 5.1%	Med-device
Lee et al [9]	Modern	CNN	PPG signal	Train or test split	349 of PPG data samples: Training: 279 sets Testing: 70 sets	CNN	Clarke error grid: 84.29%	Med-device
Shrestha et al [30]	Classical	SVM^s^	NA	NA	NA	SVM	Accuracy: 97%	Med-device
Li et al [31]	Classical	RF	NIR signal	NA	NA	RF	MAE: 17.27% Clarke error grid: 56.52%	Med-device

^a^FGSVR: fine Gaussian support vector regression.

^b^SVR: support vector regression.

^c^PPG: photoplethysmography.

^d^mARD: mean absolute relative difference.

^e^RMSE: root mean square error.

^f^LSTM: long short term memory.

^g^BG: blood glucose.

^h^CGM: continuous glucose monitoring.

^i^ND: not defined.

^j^ANN: artificial neural network.

^k^MAE: mean absolute error.

^l^CNN: convolutional neural network.

^m^GSR: galvanic skin response.

^n^NA: not applicable.

^o^MSE: mean square error.

^p^MPR: multiple polynomial regression.

^q^NIR: near-infrared.

^r^RF: random forest.

^s^SVM: support vector machine.

## Discussion

### Principal Findings

ML for BG forecasting using WDs holds great promise. Most of the studies reported RMSE and CGE for evaluation purposes, with only a couple of studies reporting high accuracy as a metric. Support vector machine algorithm was reported in 1 study with up to 97% accuracy. The general quality of the studies was considered high, as revealed by the QUADAS-2 assessment tool. The patient selection category was deemed low in half the studies, largely due to an inappropriate sampling process for selecting diverse participants among different subgroups. There were also no real applicability concerns in the quality assessment of the majority of the studies, except in the patient selection domain. The features extracted reflect the current situation as to the technologies that are commercial products, but also identify what the future holds with many prototypes in the included studies. This field is very much in its infancy, but we hereby provide insight for researchers with our findings.

### Strengths

This review followed the PRISMA extension for systematic reviews, and the protocol was preapproved by International Prospective Register of Systematic Reviews. The authors believe that by providing the quality assessment aspect, this is the first in-depth review of its kind focusing on WDs targeting BG level forecasting for diabetes using AI techniques. Compared to previous reviews, we consider our list of extracted features to be exhaustive in this field. The authors consisted of experts in the research computer science field as well as medical research practitioners, which allowed the exploration of current technologies in detail. As a result, this review reports high-impact findings to help identify gaps in the research community. The most popular databases were searched within the IT and health care fields, with further searches in Google Scholar and forward and backward reference list checking, thus reducing the risk of publication bias by allowing an exhaustive search of the literature.

### Limitations

A traditional meta-analysis was not possible due to the paucity of raw data required to meta-analyze evaluation metrics. Furthermore, there was considerable clinical and methodological heterogeneity in the included studies. Studies in the English language published between 2015 and 2021 were included; therefore, there is the possibility that some relevant studies were overlooked. Devices that could not be classified as WDs were excluded, such as electroencephalogram and electrocardiogram machines limited to hospital settings. The focus was on AI; therefore, studies that only had a statistical measurement, which is not considered an AI approach, were excluded. WD brands within our keyword searches, such as Fitbit and Apple Watch, were not included as this would return too many irrelevant results; this is in line with previous review search strategies. However, as a result, some relevant studies may have been missed in the search.

### Practical and Research Implications

There are several practical and research implications for this work. For practical implications, we find that noninvasive methods for calculating BG levels for people with diabetes to forecast BG levels are a much-welcomed advancement in this field. The ability to have such sensors on WDs that can be both stylish and fashionable, the ability to be paired with other smart devices, and general connectivity to clouds allows for continuous collection of data using many biosensors. This allows the measurement of vitals and biosignals without user interference; all of these reasons allow for a wider acceptance than existing traditional approaches such as continuous glucose monitoring. Despite the fact that there have been many studies published using WDs for diabetes, we found a lack of those that reported usage of ML and only a handful used for the purpose of BG forecasting. Although the number of studies reported in this paper is small, there is great promise due to the general quality assessment, including the accuracy levels of the ML approaches used at high levels. We see that a lot of studies are still developing their prototypes, whereas the existing commercially available devices that have already been thoroughly tested for usability and are already popular products on the market can easily be repurposed. Commercial devices are waiting to be validated with ML applications by researchers and reported in scientific journals; a quick search on retail sites reveals many commercial devices that claim to measure BG levels but have no associated studies. Adapting these existing devices would instill consumer confidence if engineers and data scientists came together and further validated these devices by reporting their effectiveness when ML techniques are applied to the generated data. Currently, there is no standard way studies are reporting performance and accuracy. Even when papers report high accuracy, this can be misleading, as from a clinical perspective, it is not important when BG levels are normal or around the normal band; the real applicability of the algorithm is in the glycemic event range. Therefore, the accuracy of these algorithms needs to be measured and reported in the range where it matters. Studies need to report these findings and not just average accuracies, providing readers with more clinically meaningful metrics. We feel it is high time that devices often classified as WDs, such as continuous glucose monitoring, which are still semi-invasive, should be less of a focus, and that studies should now focus on completely noninvasive devices, such as commercially available smart watches that make use of noninvasive sensors. There are also many opportunities within the IoT field; again, we feel there could be more integration of WDs used for BG monitoring with existing technologies and ML with Alexa, Google Homes, and Apple Watches. This could allow endless opportunities for gathering data from multiple sensors in real-time and personal patient data. Of course, the issue of privacy and data sovereignty needs to be taken seriously when it comes to mass data storage on cloud-based systems and the various interconnected devices, hospital datacenters, and consent legal and moral obligations. A multidisciplinary effort from medical practitioners, engineers, and legal experts is needed.

### Conclusions

A comprehensive systematic review, including quality assessment looking at WDs for BG level forecasting using AI and WDs, is presented following PRISMA guidelines and the QUADAS-2 tool for quality assessment.

Despite the low study numbers reported, we see great promise due to the general quality assessment, including the high accuracy levels of the ML approaches used. There is a large scope for further quality studies in this field.

The research community needs to differentiate between forecasting (based on past observations) and prediction (taking associated data, such as diet, activity, and medications along with a previous BG value, into consideration). For this study, we did not categorize studies based on the difference in definitions of these 2 terms as we found them used interchangeably in the reviewed studies.

While there are commercial-grade WDs, to the best of our knowledge, none of these devices have undergone safety and efficacy trials to be classified as medical-grade (FDA or CE), thereby currently limiting their use in clinical decision support. For example, insulin pumps, especially for patients with type 1 diabetes, require BG devices to be safe to ensure automated delivery of proper insulin dosage. However, WDs hold real promise, largely due to the broader consumer acceptance of commercially available devices coupled with their noninvasive sensors allowing for BG forecasting, which have been used using ML approaches for patients with diabetes. These WDs are getting better over time due to (1) the development of more accurate noninvasive sensors and (2) improved ML algorithms that not only use past BG values (forecasting) but also consider other information such as activity, sleep, and BMI that are also actively being measured by collocated sensors for better prediction.

Researchers have an opportunity to perform studies and validation on commercially available devices. This field is very much in its infancy, but we hereby provide insight for researchers with our findings.

We envisage the elimination of invasive devices due to WDs, but for this to happen, commercial WD manufacturers need to make raw data available as opposed to black box outputs calculating diabetes-related parameters. For example, major players currently do not provide raw PPG signals despite using PPG or NIR sensors, with the exception of devices such as Empatica; this restricts research studies to validate and optimize parameters related to glucose management and BP against traditional measurements.

## Appendix

Multimedia Appendix 1 Feature extraction description.

Multimedia Appendix 2 Risk of bias assessment.

Multimedia Appendix 3 Concerns of applicability.

## Abbreviations

AI: artificial intelligence
BG: blood glucose
CGE: Clarke grid error
ML: machine learning
NIR: near-infrared
PPG: photoplethysmography
PRISMA: Preferred Reporting Items for Systematic Reviews and Meta-Analyses
QUADAS-2: Quality Assessment of Diagnostic Accuracy Studies-2
RMSE: root mean square error
WD: wearable device

## Data Availability

All the data generated or analyzed during this study (raw data) can be made available to a reader upon reasonable request to the corresponding author.
