# Supplementary material for: The Effectiveness of Wearable Devices Using Artificial Intelligence for Blood Glucose Level Forecasting or Prediction: Systematic Review
Source: J Med Internet Res. 2023 Mar 14;25:e40259. doi: 10.2196/40259 (PMC10131991; doi:10.2196/40259)
Supplement: Multimedia Appendix 1 [file jmir_v25i1e40259_app1.docx]

**Multimedia Appendix 1.** Feature extraction description.

| **Features Name** | **Description** | **Example** |
| --- | --- | --- |
| **Wearable technology status** | Is the wearable device a prototype or is already available commercial device (Fitbit, apple watch) | Prototype, Commercial |
| **Wearable Device Type** | What type of device it is | smart band, smart watch, smart glasses, smart clothes etc |
| **Placement of wearable device** | Where the wearable device is worn during the case study/experiment in paper or normally | hands, ankles, forehead, forearms, eyes, fingers etc. |
| **Device Technology Brand** | What's the specific brand of wearable device used in study. | Fitbit, mi band, apple watch, Digi-Walker Pedometer etc |
| **Gateway** | The intermediate link between the wearable and the host device | Smartphone, Personal Computer |
| **Host device** | What is the end gate device the wearable is synchronised with | mobile, tablet, iPad, laptop, computer. |
| **Mode of Data transfer** | How data is transferred from wearable device to host device | Wi-Fi, Bluetooth, wired, GPS, Airdrop, mobile data |
| **Number of Devices** | What are the number of wearable devices used to carry out the study | Smartwatch, smart glasses |
| **Sensing Approach** | How data is being collected by the wearables with or without knowledge of user | participatory approach (manually and nonintrusive), opportunistic approach (automatic mechanisms for collecting data). |
| **Sensing Technology** | The type of sensor(s) used to capture data embedded within the wearables | photosensors, ACC (acceleration), Gyroscope (angular velocity), NIR (Near infrared) etc |
| **ML Category** | What category of ML does the model used in the study falls under | Classical, Modern |
| **AI/ML Algorithms used for forecasting** | What AI or ML algorithms are used for forecasting/prediction | Machine Learning and Ensemble Learning, Convolutional Neural Network, Long Short-term Memory etc. |
| **Input** | What type of data is fed into the models for further processing | PPG signals, fingertip images, blood glucose |
| **Data Validation Method** | What method of validation is carried out on data | K-fold cross validation, Train/Test split |
| **Data decomposition** | What percentage/size of data samples were used for training and testing/validation of models |  |
| **Algorithm Best performed** | What algorithm best performed as compared to all other used in study |  |
| **Reported Best Diagnostic performance of Model:** | The best performance models based on wearable devices data used for blood glucose forecasting only are included. All evaluation metrics calculated are extracted for that model. |  |
| **Ground Truth** | The methods that are used to gather the reference blood glucose to evaluate the performance of models in study | Med-device (portable device used in routine already for BG measurement), medical (laboratory blood test/ medical examination), expert (annotations made by experts) |
| **Number of participants** | How many participants used in the experiment – clearly specify the healthy, unhealthy, mail, female separately |  |
| **Age range of participants** | What age of participants are used for investigation in study |  |
| **Study conducted Duration** | How long the participants wore wearables to collect study data |  |
