# Supplementary material for: The Effectiveness of Wearable Devices Using Artificial Intelligence for Blood Glucose Level Forecasting or Prediction: Systematic Review
Source: J Med Internet Res. 2023 Mar 14;25:e40259. doi: 10.2196/40259 (PMC10131991; doi:10.2196/40259)
Supplement: Multimedia Appendix 3 [file jmir_v25i1e40259_app3.docx]

**Multimedia Appendix 3.** Concerns of applicability.

| Study ID | Patient Selection | Index Test | Reference Standard |
| --- | --- | --- | --- |
| Hina et al | low | low | low |
| Alfian et al | low | low | low |
| Alarcon-Paredes et al | low | low | low |
| Islam et al | low | low | low |
| Kularathne et al | high | high | high |
| Joshi et al | low | low | low |
| Zhou et al | low | low | low |
| Mahmud et al | low | low | low |
| Bent et al | low | low | low |
| Lee et al | high | low | low |
| Shrestha et al | high | low | unclear |
| Zheng Li et al | high | low | low |
